# Reduction of Glare Discomfort and Photostress Recovery Time Through the Use of a High-Energy Visible–Filtering Contact Lens

**DOI:** 10.1097/ICL.0000000000000935

**Published:** 2022-09-07

**Authors:** Lisa M. Renzi-Hammond, John Buch, Jie Xu, Billy R. Hammond

**Affiliations:** Department of Health Promotion and Behavior (L.M.R.-H.), Institute of Gerontology, University of Georgia, Athens, GA; and Research & Development, Johnson & Johnson Vision Care Inc. (J.B., J.X.), Jacksonville, FL; and Vision Sciences Laboratory, Behavioral and Brain Sciences Program, University of Georgia (B.R.H.), Athens, GA.

**Keywords:** Contact lenses, Glare discomfort, Photostress recovery, Psychophysics

## Abstract

Glare discomfort (GDC) is the slight pain (discomfort) that arises when exposed to light that exceeds one's adaptive state. Such light can also cause a temporary loss in visual function (photostress, PS). We tested the hypothesis that filtering with a high-energy visible (HEV) light–filtering contact lens can reduce GDC and speed PS recovery time.

**Methods:**

Sixty-one subjects were randomized and fit with study lenses and 58 subjects completed as cohort (20–65 years of age). A double-masked, randomized, contralateral design was used (HEV filter on one eye; control lens on the other). Participants were given a 5-s exposure to a broadband white photostressor. Video images were analyzed, and palpebral fissure size during exposure was measured, as was PS recovery time to a 2-degree mid-wave target.

**Results:**

The HEV-filtering test lens was statistically superior (*P*<0.0001) to the clear comparison contact lens with respect to the magnitude of squint (44.9% squint reduction) and photostress recovery time (24.3% faster recovery).

**Conclusions:**

High-energy visible light–filtering contacts can reduce GDC and speed PS recovery. Filtering HEV light before it is incident upon the retina is a natural strategy (e.g., by the lens and macular pigment) for attenuating some of the deleterious effects of bright broadband light.

Glare is characterized either as a loss of visual function (glare disability) or as significant visual discomfort (glare discomfort^1^). Glare discomfort (GDC) can be challenging to measure, and most of the measures are presently in use focus on quantifying aversion. These include questionnaire assessment (e.g., the De Boer scale^2^), blink rate, and squinting as assessed by measuring the diameter of the palpebral fissure or the reaction of the orbicularis muscles.^3^ Murray et al.^4^ noted, for instance, that “discomfort glare is always accompanied by a strong contraction or spasm in the muscles surrounding the eye.”

Numerous factors have been shown to influence how uncomfortable a light will be to view. These include (1) adaptive state, (2) spectral composition of the glare source, (3) luminance of the background and glare source, (4) spatial properties of the glare source, (5) angle of the glare source with respect to the line of sight, and (6) time of day (see Pierson et al.^5^ for a more comprehensive list). These same factors also tend to drive how “blinding” a light will appear.^6^ When exposed to a sufficiently bright light, an individual will temporarily lose the ability to see objects obscured by that light, even when the exposure ends. The time necessary to see again is referred to as photostress recovery (PSR) time.^7^

The experiences of GDC and photostress (PS) tend to depend upon the ecological significance of the test stimuli that induce them. For example, to assess the real-world impact of GDC and PSR, studies often go to some effort to match the visual stimuli in their experiment to those an individual would likely encounter in realistic settings (reviewed by Pitts^8^). When this has been done (e.g., matching the glare source to emulated sunlight^9^), it becomes clear that humans have evolved some natural means for moderating light stress.^10^ This often takes the form of intraocular filtering. For instance, both the blue-absorbing retinal macular pigments^11^ and ocular melanin^12^ have been shown to attenuate GDC. It is little wonder that filtering sunglasses (e.g., photochromic spectacles^13^) emerged as a common means of decreasing GDC and PS.

In this study, we compared GDC and PSR time in subjects wearing a high-energy visible (HEV, typically defined as absorbing light from 380 to 500 nm^14^) light-filtering test contact lens in one eye compared with a relatively clear control contact lens in the other (a contralateral design). Figure 1 shows the spectral transmission of the study lenses. Glare discomfort was quantified as the magnitude of squint elicited with bright light exposure, and PSR was measured as the amount of time necessary to recover visual function after such exposure.

**FIG. 1.**
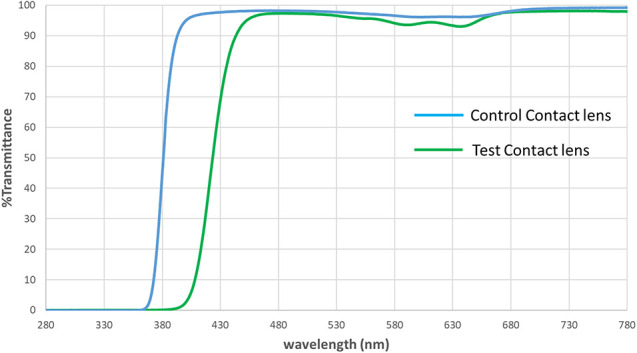
Spectral transmission of the study contact lenses.

## METHODS

### Subjects

**FIG. 2.**
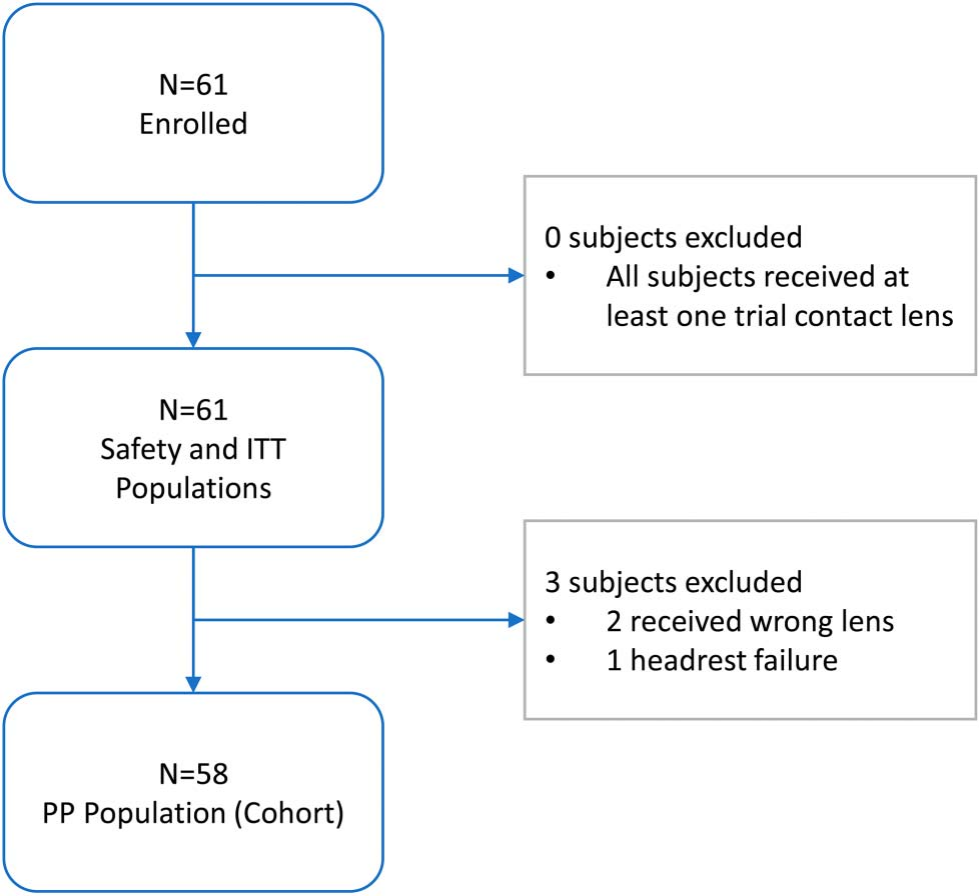
Subject accountability.

A total of 61 subjects were randomized (intent-to-treat [ITT] subjects), fit with a study lens, and tested at a single clinical site (Georgia Center for Sight, Greensboro, GA). Among the 61 subjects, ages ranged from 20 to 65 years (*M*=39.60±12.21 years). Most (77%) participants identified as female, and the remaining participants (23%) identified as male. A total of 31.1% of participants identified as black or African American; 68.9% of participants identified as white/caucasian. A total of 3.3% of participants identified as ewhite/Hispanic. All participants were habitual contact lens wearers; either senofilcon A or other spherical silicone hydrogel soft contact lenses with best-corrected visual acuity of 20/25 or better in each eye (−1.00 to −4.50 diopters). A basic clinical examination was used to exclude any overt ocular abnormality (no subjects needed to be excluded). Subject accountability is shown in Figure 2.

#### Ethics

The study was performed in accordance with “Clinical investigation of medical devices for human subjects” (with ISO 14155:2011) and followed the tenets of the Declaration of Helsinki. Written and verbal informed consent were obtained from all subjects. The protocols were approved by the Sterling Institutional Review Board, Atlanta, GA.

#### Lens Assignment/Masking

This was a prospective, randomized, controlled, double-masked, contralateral study. Subjects were randomly assigned to one of two conditions: test lens OS, control lens OD; or test lens OD, control lens OS. The clinician fitting the lenses was unmasked, but subjects were unaware of the identity of the lenses, and the investigator conducting the psychophysical testing was also not informed about which eye had which lens (the two lens types are not visually distinguishable on eye). Instead, lens type was identified by code only, and the link between the code and the lens type was not revealed to the investigator conducting the psychophysical testing until after the database had been locked. Lens powers were selected such that the spherical overrefraction was plano in the right and left eyes.

### Apparatus and Procedure

The light for the stimuli was produced by a 1000-Watt xenon arc source with a housing that was modified to allow dual-channel exit (Newport Optics, Irvine, CA); one channel was used to form the photostressor and the other to form a central grating target presented in Maxwellian view with the contralateral eye patched (for further details see Renzi-Hammond et al.^15^). Alignment of the subject's eye with the optical system was maintained with a forehead rest and a dental impression bite bar that was custom-fit for each participant. An auxiliary optical channel with a high-resolution camera, infrared illumination, and monitor was used to monitor the pupil during testing to ensure proper fixation and sustained alignment, and was used, along with biometric software (Amscope, Irvine, CA) to measure GDC.

All photometric calibrations (both in the visible and ultraviolet) were performed using an ILT950 spectroradiometer (International Light Technologies, Peabody MA). Wedge and neutral density radiometric calibrations were performed using a Graseby Optronics United Detection Technology instrument (Orlando, FL). The same instrument was used periodically to ensure that the total light output of the optical system remained consistent throughout the study.

#### The Test Target

The visual target was used to test for the recovery of vision after the PS exposure. The visual target was a 2°-diameter circular grating (square wave, 16 lines/inch) with a wavelength composition that peaked at 580 nm (32 nm full bandpass). This target was shuttered with one-second exposures separated by one-second delays, to prevent adaptation to the target.

#### Photostressor

The second channel was used to produce a 16.25-degree background field for GDC and PSR time testing. The xenon light that was used for this measurement has a broadband emission spectrum with a chromaticity of u'=0.20, v'=0.46 (CCT=6,824) that emulated sunlight. The relatively large circular test light was presented at high intensity (4.7 log Trolands) for 5 seconds.

#### Procedure

Before each test day, a ruler was secured in the headrest assembly and aligned to the focal point for light exiting the optical system. The camera was then brought into focus with the ruler and light focal point in the center of the viewing screen. The biometric software was used to measure the number of pixels that corresponded with a fixed distance on the ruler (12.7 mm), to enable translation from pixels to millimeters in still images used for analysis. The participant was then aligned to the same position as the ruler, thus placing the stimulus in Maxwellian view and the test eye in optimal focus for the camera.

The psychophysical procedure was similar to that reported in the past.^14^ The recording software was started while the participant viewed the test target in absence of the photostressor. Once the investigator confirmed that the participant could clearly see the target, the investigator introduced the photostressor by actuating the programmable shutter for a fixed, five-second increment. As soon as the shutter closed, thus removing the photostressor, the participant was asked to press a buzzer once they regained sight of the target. Once the participant pressed the buzzer, the recording was stopped, and the time needed to regain sight of the target was recorded as PSR time. The participant was then asked to sit with their eyes closed for a one-minute wash-out period. After the one-minute wash out, participants were asked to confirm that no afterimages from the photostressor remained, and the procedure was repeated. Two trials were completed per eye, with the starting eye randomized.

Once the trials were complete, a single trained investigator masked to lens identity analyzed the video. The raw.mp4 file generated during the recording was opened in QuickTime, using a MacBook Pro laptop computer connected to the camera system. The investigator partitioned the video into still frames and captured two frames for analysis: the frame with the subject's eye open naturally before the onset of the photostressor, and the frame that yielded maximum squint in the presence of the photostressor. The frames were then exported into the Amscope biometric software program that accompanied the camera, and the investigator measured the size of the palpebral fissure as the distance (number of pixels) between the top and bottom lids, in both conditions. The pixel values were then translated into millimeter of squint (GDC), which were averaged between the two trials for each eye/lens type.

### Statistical Analysis

Statistical analyses were performed using SAS version 9.4. The ITT method was applied to test statistical superiority.^16^

The analyses of GDC and PRT were performed separately using a generalized linear mixed model (GLMM) with a lognormal distribution that appropriately specified the covariance structure of the residual errors from within-subject repeated measures taken from different eyes. The models were adjusted for subject's age, dominant eye, gender, race, iris category (dark iris and light iris), and baseline self-reported light sensitivity score (on a 0–100 scale). Interaction effects between lens and dominant eye/iris category were included as fixed effects when statistically appropriate. The estimated least squares means of the log-transformed observations were transformed back to the original scale using the exponential function. With this back transformation, estimates of the median squint magnitude and time to recovery on the original scale was derived for each study lens. The improvement percent (i.e., a percentage decrease relative to the control lens) was derived based on the model estimated medians and tested for statistical superiority of the test lens compared with the control.

## RESULTS

### Glare Discomfort

**FIG. 3.**
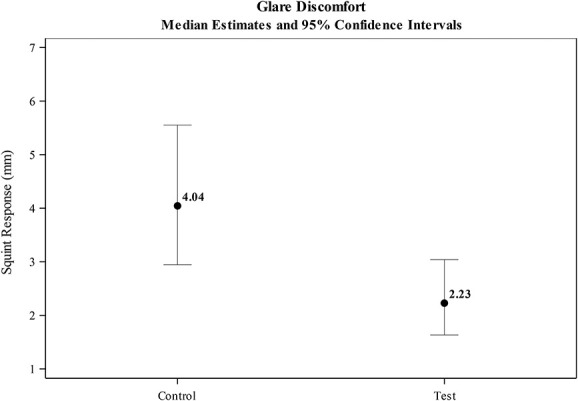
Estimated discomfort glare (squint response in millimeters).

**FIG. 4.**
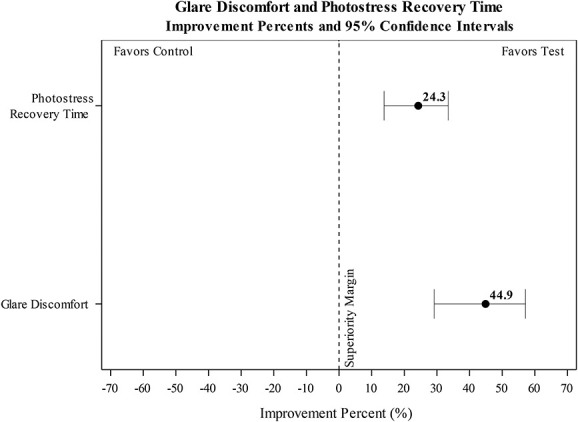
Estimated improvement percent for PSR time and GDC.

The estimated magnitude of the squint response (as the difference in palpebral fissure size when the eye was open naturally, vs. the palpebral fissure size when the eye was exposed to the photostressor) was significantly smaller in test eyes fitted with the HEV light–filtering contact lens (2.23 mm), compared with the control lens (4.04 mm; see Fig. 3 and Table 1). Test eyes wearing the HEV light–filtering contact lens experienced a 44.9% reduction in squint, compared with test eyes wearing the clear control lens (95% CI=29.2, 57.1; *P*<0.0001; see Fig. 4).

**TABLE 1.** Descriptive Statistics and Results of Statistical Tests

	Test (N=61)	Control (N=61)
Glare discomfort (mm)		
Mean (SD)	3.13 (2.014)	5.00 (2.906)
Median	3.05	4.49
Model estimated median	2.23, 95% CI=(1.63, 3.04)	4.04, 95% CI=(2.95, 5.55)
Model estimated improvement percent (%)	44.9, 95% CI=(29.2, 57.1), *P* value <0.0001	
Photostress recovery time (seconds)		
Mean (SD)	9.20 (6.847)	11.98 (9.961)
Median	8.22	9.34
Model estimated median	5.45, 95% CI=(4.31, 6.88)	7.19, 95% CI=(5.66, 9.14)
Model estimated improvement percent (%)	24.3, 95% CI=(13.8, 33.5), *P* value <0.0001	

N, total number of subjects; CI, confidence interval.

### Photostress Recovery Time

**FIG. 5.**
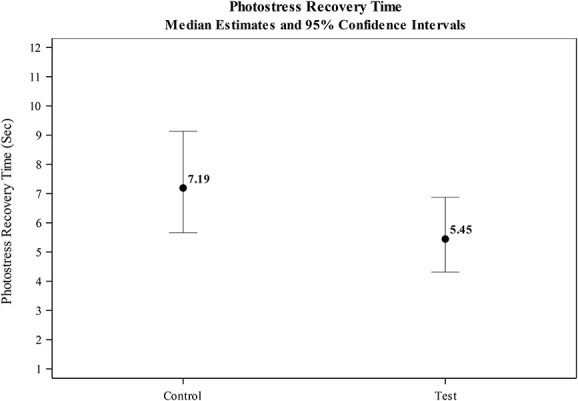
Estimated photostress recovery time (seconds).

Median time to recovery estimated from the GLMM analysis was significantly shorter for eyes wearing the HEV light–filtering contact lens (5.45 s), compared with the clear control lens (7.19 s; see Table 1 and Fig. 5). Test eyes wearing the HEV light–filtering contact lens experienced a 24.3% reduction in recovery time (95% CI=13.8, 33.5; *P*<0.0001; see Fig. 3).

## DISCUSSION

Many accidents occur as a result of loss in visual function.^17^ The Centers for Disease Control and Prevention have ranked unintentional injuries (accidents) as the third leading cause of death in the United States (approximately 173,040) per year.^18^ Redelmeier and Raza^19^ showed that the risk of a life-threatening car accident was highest during the day and was increased by 16% because of the glare induced by bright sunlight. In this study, we measured two types of visual response to exposures of bright emulated sunlight: squinting and temporary loss of visual function. An eye with an HEV light–filtering contact lens was worn in one eye, and a relatively clear contact lens was worn in the fellow eye. We found that squinting was reduced and PSR times were shortened in the eye with the HEV light–filtering contact lens. Both the subject and experimenter were masked as to which eye contained the test or comparison lens. To the degree that such differences measured in the laboratory translate to real-world improvements in vision (a major goal in the design of our experiments is to maximize their ecological validity), the potential for contact lens design to improve vision under certain circumstances (ranging from driving safety to athletic performance) is significant.

The optical density of the HEV light–filtering contact lens that we tested asymptotes at approximately 460 nm with its strongest visible absorbance between approximately 380 and 440 nm (average optical density=1.02). However, the effects on squinting (approximately 45% comparative reduction) and PSR (24%) were surprisingly strong. This finding suggests that filtering these very short wavelengths may be particularly useful in reducing some of the deleterious effects of bright light without simultaneously influencing peak spectral sensitivity (reducing the strongly used portion of the visible spectrum). This is consistent with data showing that, all things equal, discomfort is exaggerated at the lowest wavelengths.

In 2003, Stringham et al.^20^ used a criterion response to measure an action spectrum for GDC (photophobia) (see also Flannagan et al.^21^). Glare discomfort was defined as squinting in response to exposure to narrow band wavelengths of light. The intensity of the light was increased until the exposure elicited a predefined level of squint. This amount of squinting (measured with electromyography) was the criterion response, and it was kept constant while the intensity of each waveband of light was varied. The main finding, strongly linear, was that less energy was required at shorter wavelengths to elicit the criterion response than at longer wavelengths. This effect was predictable based on known light damage curves (e.g., Ham et al.^22^), which led to the interpretation by the authors that squinting evolved as a protective mechanism against actinic light. Taken together with our results, filtering HEV light seems to be particularly efficacious in reducing some of the more deleterious effects of bright light exposures.
